# P-1972. SARS-CoV-2 Vaccine–Induced IgG4 Response Negatively Correlates with Neutralizing Antibodies and Fc Effector Functions

**DOI:** 10.1093/ofid/ofae631.2130

**Published:** 2025-01-29

**Authors:** Raj Kalkeri, Mingzhu Zhu, Shane Cloney-Clark, Anand Parekh, Drew Gorinson, Rongman Cai, Soham Mahato, Pradhipa Ramanathan, L Carissa Aurelia, Kevin J Selva, Anthony M Marchese, Louis F Fries, Lisa M Dunkle, Amy W Chung, Joyce S Plested

**Affiliations:** Novavax, Inc., Gaithersburg, Maryland; Novavax, Gaithersburg, Maryland; Novavax, Gaithersburg, Maryland; Novavax, Inc., Gaithersburg, Maryland; Novavax, Inc., Gaithersburg, Maryland; Novavax, Inc., Gaithersburg, Maryland; Novavax, Inc., Gaithersburg, Maryland; Doherty Institute, Melbourne, Victoria, Australia; Department of Microbiology and Immunology, University of Melbourne, at the Peter Doherty Institute for Infection and Immunity, Victoria, 3000, Australia, Melbourne, Victoria, Australia; Doherty Institute, Uni of Melbourne, Melbourne, Victoria, Australia; Novavax, Inc., Gaithersburg, Maryland; Novavax, Inc., Gaithersburg, Maryland; Novavax, Inc., Gaithersburg, Maryland; University of Melbourne, Melbourne, Victoria, Australia; Novavax, Gaithersburg, Maryland

## Abstract

**Background:**

IgG4, the least abundant human IgG subtype, increases after repetitive exposure to some antigens, and may induce immune tolerance. Studies have found that repeat mRNA SARS-CoV-2 vaccination leads to large proportional increases in spike (S)-specific IgG4. By contrast, increased IgG4 has not been observed following repeat vaccination with recombinant SARS-CoV-2 S (rS) protein (NVX-CoV2373, Novavax). Whether vaccine-induced anti-S IgG4 might impair SARS-CoV-2 immunity remains unknown.

**Figure:**
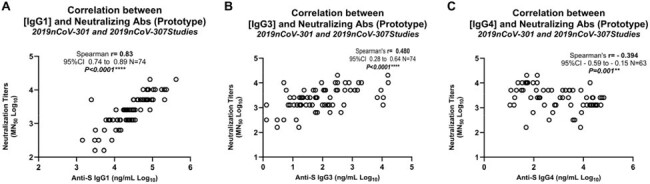
Serum anti-Spike IgG1 (A), IgG3 (B), and IgG4 (C) concentrations (ng/mL) measured by ELISA were correlated with neutralizing antibody titers (MN50) measured by infectious virus microneutralization assay.

**Methods:**

Sera were collected from study participants in 2019nCoV-307 (NCT05463068) and 2019nCoV-301 (NCT04611802), including 2 groups from 2019nCoV-307 who received 3 homologous mRNA doses followed by 1 NVX-CoV2373 dose (mRNA-1273, Moderna, n=10; or BNT162b2, Pfizer, n=10), and a 3^rd^ group from 2019nCoV-301 who received 4 homologous NVX-CoV2373 doses (n=18). Sera collected ≥6 months after the last dose in the first 2 groups and ∼4 weeks after the last dose in the 3rd group were assessed for neutralizing antibodies (nAb), IgG subclass profiles (anti-S total IgG, IgG1, IgG2, IgG3, and IgG4), and Fc effector activities (cell-based ADCP assay; surrogate ADCC by FcγRIIIa binding; and ADCD by C1q binding) after repeated NVX-CoV2373 or a single NVX-CoV2373 dose following repeated mRNA vaccine.

**Table:**
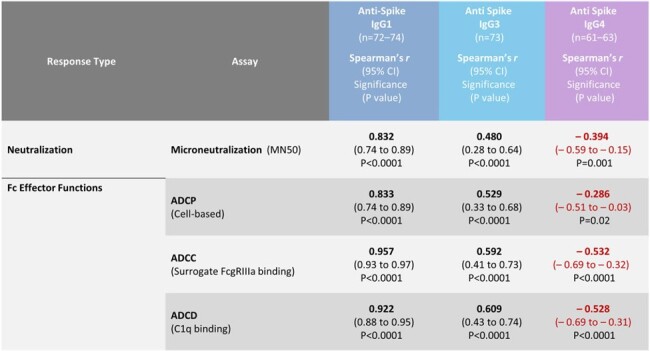
Summary of correlations between IgG1, IgG3, and IgG4 with neutralizing antibody titers (ancestral) and Fc effector functions in 2019nCoV-301 and 2019nCoV-307 studies.

**Results:**

Increased nAb titers after NVX-CoV2373 were observed irrespective of the prior vaccine background, whereas increased IgG4 levels were only observed in recipients of mRNA vaccines. Spearman's rank correlation between nAb and Fc functions found significant positive correlations with anti-S IgG1 and IgG3 (Figure A, B), and significant negative correlation with anti-S IgG4 (Figure C) antibody responses (Table).

**Conclusion:**

These are the first data to demonstrate the negative correlation of increased SARS-CoV-2 vaccine–induced anti-S IgG4 levels and nAb titers and Fc effector functions. The limitations of our data include small sample sizes, and potential differences attributable to vaccination and serum collection intervals. IgG4-mediated suboptimal nAb and reduced Fc effector function could reduce the vaccine effectiveness of the SARS-CoV-2 response, though more work is needed to understand its impact on the optimization of vaccine platform choice.

**Disclosures:**

Raj Kalkeri, PhD, Novavax: Employee|Novavax: Stocks/Bonds (Public Company) Mingzhu Zhu, n/a, Novavax, Inc.: employee|Novavax, Inc.: Stocks/Bonds (Public Company) Shane Cloney-Clark, n/a, Novavax, Inc.: employee|Novavax, Inc.: Stocks/Bonds (Public Company) Anand Parekh, M.Sc., Novavax, Inc.: employee|Novavax, Inc.: Stocks/Bonds (Public Company) Drew Gorinson, B.S., Novavax, Inc.: employee|Novavax, Inc.: Stocks/Bonds (Public Company) Rongman Cai, PhD, Novavax, Inc.: employee|Novavax, Inc.: Stocks/Bonds (Public Company) Soham Mahato, PhD, Novavax, Inc.: employee|Novavax, Inc.: Stocks/Bonds (Public Company) Anthony M. Marchese, PhD, Novavax Inc: Employee|Novavax Inc: Stocks/Bonds (Public Company) Louis F. Fries, III, MD, Novavax, Inc.: contractor for Novavax|Novavax, Inc.: Stocks/Bonds (Public Company) Lisa M. Dunkle, MD, Novavax, Inc: Employee|Novavax, Inc: Ownership Interest|Novavax, Inc: Stocks/Bonds (Public Company) Amy W. Chung, PhD, Novavax: Grant/Research Support Joyce S. Plested, n/a, Novavax, Inc.: employee|Novavax, Inc.: Stocks/Bonds (Public Company)

